# Oxazolo[5,4-*d*]pyrimidines as Anticancer Agents: A Comprehensive Review of the Literature Focusing on SAR Analysis

**DOI:** 10.3390/molecules30030666

**Published:** 2025-02-03

**Authors:** Aleksandra Sochacka-Ćwikła, Marcin Mączyński

**Affiliations:** Department of Organic Chemistry and Drug Technology, Faculty of Pharmacy, Wroclaw Medical University, 211A Borowska Street, 50-556 Wroclaw, Poland

**Keywords:** oxazolo[5,4-*d*]pyrimidines, anticancer activity, SAR analysis

## Abstract

Oxazolo[5,4-*d*]pyrimidines have been found to exhibit a wide range of biological activities, including the inhibition of various enzymes and signaling pathways associated with carcinogenesis. The objective of this review is to demonstrate that the oxazolo[5,4-*d*]pyrimidine scaffold represents a valuable structure for the design of novel anticancer therapies. The article provides a comprehensive overview of the chemical structure and pharmacological properties of oxazolo[5,4-*d*]pyrimidine derivatives, drawing upon the literature and international patents from 1974 until the present. Notably, the review explores structure–activity relationships (SAR) with a view to enhancing the therapeutic efficacy of oxazolo[5,4-*d*]pyrimidines.

## 1. Introduction

Cancer represents a significant global health challenge, currently ranking as one of the leading causes of mortality worldwide. According to the World Health Organization (WHO), the number of global cancer cases and cancer-related deaths has increased in recent years. For instance, in 2022, there were nearly 20 million new cancer cases and 9.7 million cancer deaths [1]. Cancer is a group of diseases characterized by the uncontrollable growth of transformed cells that are subject to evolution by natural selection [2]. The disease may manifest in any organ or tissue of the body. The classification of cancerous tissue is dependent on the type of tissue from which it originates and the primary site. There are six major histopathological types of cancer: carcinoma, sarcoma, myeloma, leukemia, lymphoma, and mixed types. Cancerous cells have the capacity to invade surrounding tissues or to spread to more distant ones (i.e., metastasis) via the bloodstream or lymphatic system. The growth and metastasis of cancerous cells depend on angiogenesis, which is defined as the process whereby new blood vessels are formed as a consequence of signaling molecules produced by rapidly dividing cancerous cells [3].

Heterocyclic compounds are of crucial importance in the development of novel anticancer agents. In recent years, the majority of small molecule drugs to treatment hematological and solid cancers approved by the FDA have been nitrogen-containing heterocycles with an oxygen atom [4,5]. The oxazolo[5,4-*d*]pyrimidine system is a fused heterocyclic ring containing both nitrogen and oxygen atoms. It can be regarded as a purine analog, in which the imidazole ring is replaced with an oxazole ring. Purine analogs represent a class of anticancer agents known as antimetabolites, which are of great therapeutic importance. Azathioprine and thioguanine are two such analogs that have been approved by the FDA for use in cancer therapy [6]. The oxazolo[5,4-*d*]pyrimidine system represents a universal scaffold that has been employed in the design of bioactive inhibitors of enzymes such as adenosine kinase (AK) [7] and acetyl-CoA carboxylase (ACC) [8]. Oxazolo[5,4-*d*]pyrimidine derivatives have also been identified as antagonists of various receptors, including the purinergic G protein-coupled receptor (P2Y1) [9], the transient receptor potential vanilloid type 1 receptor (TRPV1) [10], and the angiotensin II receptor (ATR2) [11]. In some biological studies, they have been found as anti-angiogenic or antiviral agents. Additionally, certain oxazolo[5,4-*d*]pyrimidines have been identified as potential immunosuppressants or inhibitors of ricin and Shiga toxins [12,13]. It is noteworthy that a number of these compounds have also demonstrated anticancer activity [14].

**Figure 1:**
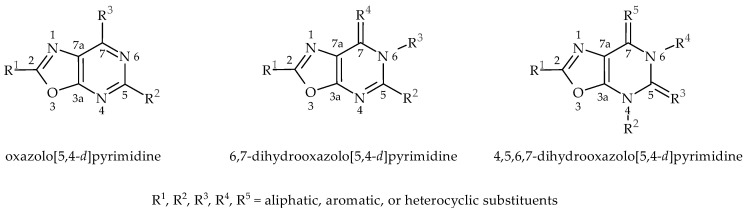
The known structures of oxazolo[5,4-*d*]pyrimidines with anticancer activity.

This article presents and discusses the anticancer agents that have been identified among the oxazolo[5,4-*d*]pyrimidine derivatives. A comprehensive search of the electronic databases SciFinder, Web of Science, Scopus, and PubMed has been conducted to collect articles focusing on oxazolo[5,4-*d*]pyrimidines and their anticancer activity (Figure 1). The present review summarizes both compounds targeting molecular structures involved in the growth and spread of cancer cells, and those with an unclear identification of mechanism of action. The aim of this study is to undertake a detailed analysis of research articles and international patents from 1974 to the present, with particular emphasis on the structure–activity relationship (SAR). The results of the SAR analysis for the corresponding series of compounds are summarized in the figures, which also contain tables presenting detailed biological data for the most potent derivatives. It is important to note that this work does not include an overview of the methods of synthesis of the oxazolo[5,4-*d*]pyrimidine system, since a systematic analysis of these methods was previously reported [15,16]. It is evident that the synthetic strategies remain unchanged, encompassing two primary pathways starting from either a functionalized pyrimidine or oxazole derivatives.

## 2. Oxazolo[5,4-*d*]pyrimidines as Anticancer Agents with Targeted Mechanism of Action

### 2.1. VEGFR2 and/or EGFR Inhibitors

Vascular endothelial growth factor receptor-2 (VEGFR-2) and epidermal growth factor receptor (EGFR) belong to the family of tyrosine kinase receptors (RTKs), which are frequently mutated and/or overexpressed in different kinds of human cancers, namely lung, breast, and colorectal carcinoma. VEGFR2 is a key factor involved in tumor angiogenesis, i.e., the process of formation of new blood vessels that supply oxygen and nutrients to proliferating cancerous cells. VEGFR2 activity leads to the activation of downstream signaling which mediates vascular permeability, as well as endothelial cell migration, proliferation, and survival. It is also responsible for the reduction of cell apoptosis and changes in cytoskeleton function [17]. Conversely, EGFR exerts critical functions in the physiology of epithelial cells, which are the precursors of all carcinomas. The paracrine loops comprising cancerous and stromal cells enable EGFR to facilitate cancer growth and metastasis [18]. Furthermore, EGFR is increasingly being acknowledged as a biomarker of resistance in cancerous cells, as its amplification or secondary mutations have been identified as occurring under the influence of pharmaceutical agents [19].

**Figure 2:**
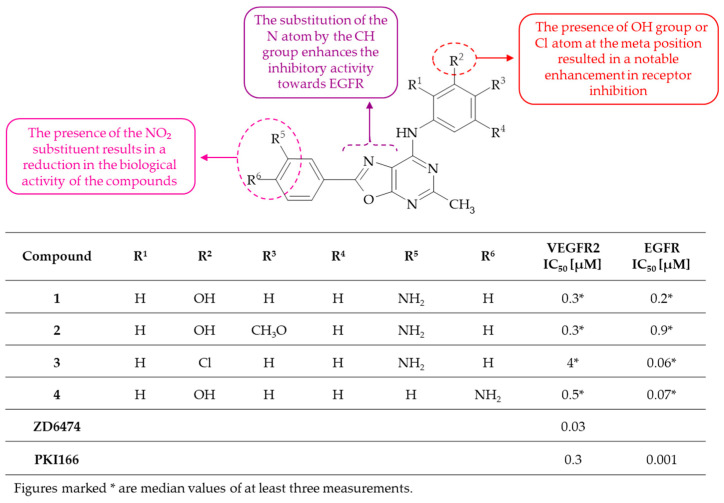
SAR study of oxazolo[5,4-*d*]pyrimidines as dual VEGFR2 and EGFR inhibitors.

The compounds with an oxazolo[5,4-*d*]pyrimidine scaffold, an aromatic ring at C(2) and an anilino moiety at C(7) were designated by Martin-Kohler et al. as VEGFR2 and EGFR inhibitors [20]. The most effective VEGFR2 inhibitors from the series were compounds **1**, identified as 3-{[2-(3-aminophenyl)oxazolo[5,4-*d*]pyrimidin-7-yl]amino}phenol, and **2**, specifically 5-{[2-(3-aminophenyl)oxazolo[5,4-*d*]pyrimidin-7-yl]amino]-2-methoxyphenol, with a half maximal inhibitory concentration (IC_50_) value of 0.3 µM. In contrast, the most potent EGFR inhibitors showed IC_50_ values 0.006 µM and 0.007 µM for compounds **3**, i.e., 3-{[2-(4-aminophenyl)oxazolo[5,4-*d*]pyrimidin-7-yl]amino}phenol and **4**, i.e., 2-(3-aminophenyl)-*N*-(3-chlorophenyl)oxazolo[5,4-*d*]pyrimidin-7-amine, respectively. It can be observed that the inhibitory activity of derivatives **1**–**4** was markedly less potent than that of the reference VEGFR2 inhibitor ZD6474 [21] and the EGFR inhibitor PKI166 [22], respectively. Nevertheless, the general structure–activity relationship can be established (Figure 2). The nitrophenyl-substituted oxazolo[5,4-*d*]pyrimidines were found to be inactive. Conversely, the substituent, such as a hydroxyl group or Cl atom, at the meta position of the anilino moiety caused an increase in receptor inhibition. The replacement of the N atom in the oxazolo moiety by the CH group, i.e., the formation of an appropriate furo[2,3-*d*]pyrimidine, proved to be beneficial for the development of more efficacious EGFR inhibitors. It is hypothesized that this is an electronic effect that is characteristic of EGFR, rendering the oxazole N atom unfavorable. An alternative hypothesis is that an intramolecular hydrogen bond between the NH group in the anilino moiety and the N atom in the oxazolo moiety is responsible for the unfavorable conformation of the molecule, which in turn impairs its ability to bind to the enzyme [20]. To summarize, the presented oxazolo[5,4-*d*]pyrimidines and, in particular, their furo[2,3-*d*]pyrimidines counterparts represent a noteworthy addition to the known classes of RTK inhibitors.

**Figure 3:**
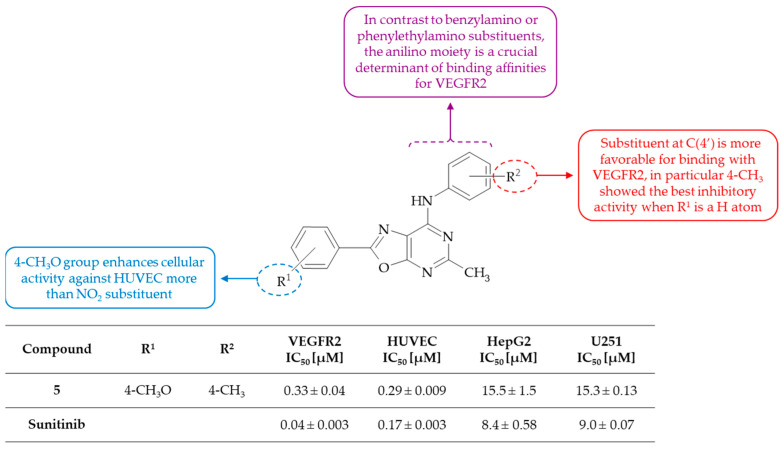
SAR study of 2,5,7-trisubstituted oxazolo[5,4-*d*]pyrimidines as inhibitors of VEGFR2 and VEGF-induced HUVEC proliferation.

Deng and coworkers identified a series of 2,5,7-trisubstituted oxazolo[5,4-*d*]pyrimidines, which were found to be potent inhibitors of VEGFR2 and VEGF-induced HUVEC proliferation [23]. The most effective inhibitor, 2-(4-methoxyphenyl)-5-methyl-*N*-(4-methylphenyl)oxazolo[5,4-*d*]pyrimidin-7-amine **5**, demonstrated IC_50_ values of 0.33 and 0.29 µM against VEGFR2 kinase and HUVEC, respectively. In silico analysis of this derivative suggested that the oxazolo[5,4-*d*]pyrimidine moiety is positioned in the ATP binding site, and formed two H-bonds with the NH group of Lys-868 and the backbone NH group of Asp-1046. Moreover, the findings of the VEGFR2 activity study indicated that a CH_3_ group in the para position of the anilino moiety demonstrated notable inhibitory potency. Furthermore, the introduction of a Cl atom in the meta position and an F atom in the para position of the phenyl ring also exhibited good potency against VEGFR2 (IC_50_ = 0.62 µM). On the other hand, the introduction of a NO_2_ group resulted in a reduction in VEGFR2 activity, suggesting that an electron-withdrawing group may not be preferred to receptor binding. The further biological evaluations against multiple kinases indicated that tested oxazolo[5,4-*d*]pyrimidines showed selective inhibitory activities against VEGFR2, VEGFR1, and EGFR (inhibitory rate > 60%) higher than against other protein kinases such as FGFR, PDGFR, Flt-3, JAK2, and KIT. Additionally, they exhibited potent inhibitory activities against cluster of differentiation 31 (CD31), indicating potent anticancer activity. The results of cytotoxic assay of this series of oxazolo[5,4-*d*]pyrimidine derivatives against VEGF-induced human umbilical vein endothelial cell (HUVEC) proliferation, and two cancer cell lines (HepG2 and U251) stated that the compounds generally showed moderate inhibitory activities against HepG2 and U251 with IC_50_ values in the range of 10^−5^–10^−6^ M (sunitinib, known VEGFR2 inhibitor [24], as positive control: 8.4 and 9.0 µM for HepG2 and U251, respectively) and good potency against HUVEC proliferation with IC_50_ values generally at 10^−7^ M. In accordance with the findings of the VEGFR2 kinase assay, the 4-CH_3_ and 4-CF_3_ substituents of the aniline moiety at C(7) of the oxazolo[5,4-*d*]pyrimidine system are responsible for the observed good inhibitory potencies (Figure 3). The substituent at the para-substituted ring is more favorable for binding with the receptor, as evidenced by a significant increase in VEGF-HUVEC inhibitory activity, from 7.98 to 1.20 µM, for the 2-chloroanilino derivative in comparison with the 4-chloroanilino derivative, respectively. Furthermore, the introduction of a benzylamino or phenylethylamino moiety to the C(7) position of the oxazolo[5,4-*d*]pyrimidine scaffold resulted in the lack of activity in the HUVEC proliferation assay. Overall, the inhibitory activities of derivatives with various substituents on the phenyl ring in the C(2) of the oxazolo[5,4-*d*]pyrimidine system were found to be comparable to those of sunitinib, except for the derivative with a NO_2_ group as substituent. Notably, the replacement of the phenyl ring at C(2) position by a methylpiperazine substituent has been observed to result in enhanced activity. This is in contrast to the effect observed when a CH_3_ group is present, which has been found to achieve unsatisfactory results. A molecular docking in the active site of VEGFR2 of **5** suggests that the oxazolo[5,4-*d*]pyrimidine moiety is positioned in the ATP binding site, and forms two H-bonds with the NH group of Lys-868 and the backbone NH group of Asp-1046. However, further modifications of these compounds based on SAR analysis are required to discover more potent novel anti-angiogenic agents.

**Figure 4:**
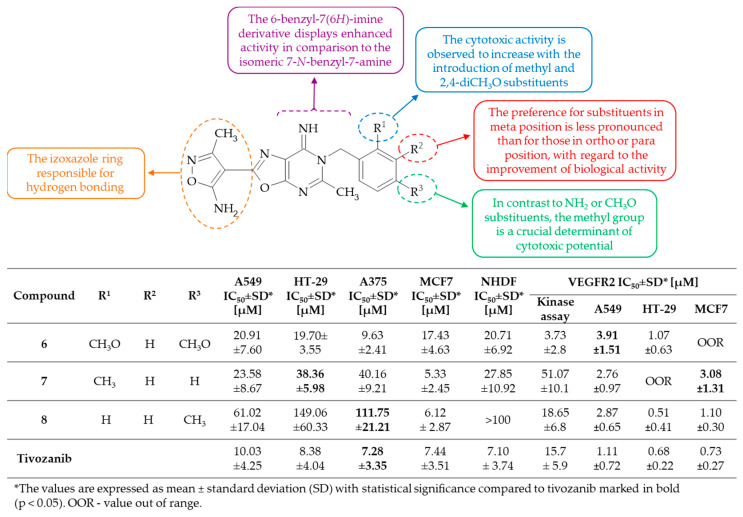
SAR study of 7-*N*-benzyloxazolo[5,4-*d*]pyrimidin-7-amine and 6-*N*-benzyloxazolo[5,4-*d*]pyrimidin-7(6*H*)-imine derivatives as VEGFR2 inhibitors and anti-angiogenic agents.

In a recent study, Sochacka-Ćwikła and colleagues synthetized the series of 6-*N*-benzyloxazolo[5,4-*d*]pyrimidin-7(6*H*)-imines and the isomeric 7-*N*-benzyl-7-amine derivative, obtained by Dimroth rearrangement [25]. It is noteworthy that the structures of the novel derivatives were designed based on the previous results of the studies conducted by Martin-Kochler et al. [20] and Deng et al. [23]. The obtained derivatives were then screened for cytotoxic activity against normal cell lines, i.e., normal human dermal fibroblasts (NHDF), and four human cancer cell lines, which were characterized by the overexpression of VEGFR2, including lung cancer (A549), colon cancer (HT-29), melanoma (A375), and breast cancer (MCF7). Tivozanib, which is a potent VEGFR2 inhibitor [26], was used as a reference drug in both in vitro experimental assays and in the molecular docking study. The most active compound **6**, i.e., 4-[6-(2,4-dimethoxybenzyl)-7-imino-5-methyl-oxazolo[5,4-*d*]pyrimidin-2-yl]-3-methyl-isoxazol-5-amine, was observed to exhibit comparable cytotoxic activity to tivozanib against all tested anticancer cell lines, while also demonstrating equal toxicity to NHDF. Derivatives **7**, i.e., 4-[6-(2-methylbenzyl)-7-imino-5-methyl-oxazolo[5,4-*d*]pyrimidin-2-yl]-3-methyl-isoxazol-5-amine, and **8**, i.e., 4-[6-(4-methylbenzyl)-7-imino-5-methyl-oxazolo[5,4-*d*]pyrimidin-2-yl]-3-methyl-isoxazol-5-amine, displayed comparable activity to tivozanib against three anticancer lines, with **8** exhibiting the additional property of no cytotoxicity against NHDF. In accordance with previous findings, the 7-*N*-benzyl-7-amine isomer was found to be devoid of activity in the cytotoxicity assay. The 6-benzyl-7(6*H*)-imine isomers were observed to exhibit a range of activities against the tested cancer cell lines, suggesting that the benzyl moiety in the 6 position of the oxazolo[5,4-*d*]pyrimidine system is essential for biological activity. The SAR analysis indicated that the 2,4-diCH_3_O substituent or the CH_3_ group, in particular in the para- or orto-position, rather than the meta position, of the benzyl moiety was beneficial for the cytotoxic potential of compounds (Figure 4). Conversely, the CH_3_O or NH_2_ group in the para position of the benzyl moiety was observed to result in a reduction in the cytotoxic activity of the derivatives. Molecular docking analysis of compound **8** revealed that the isoxazole moiety forms hydrogen bonds with the key amino acids Glu885 and Cys919 in the hinge region of the kinase domain. Additionally, the imino group of the oxazolo[5,4-*d*]pyrimidine core establishes an additional hydrogen bond with the conserved DFG domain, while the 4-methylbenzyl substituent interacts with the allosteric pocket (HYD-II region). This suggests that the binding mode of the compound is like that of type II VEGFR2 inhibitors. The result of VEGFR2 kinase activity assay indicated that only compounds **6** and **8** were identified as effective VEGFR2 inhibitors. The relatively weaker inhibitory activity against VEGFR2 observed with compound **7** in comparison to tivozanib indicated that the para-substituted benzyl moiety is of critical importance for receptor binding. Additionally, the impact of the tested derivatives on the expression of VEGFR2 in A549, HT29, and MCF-7 cell lines revealed that compound **8** was a potent inhibitor of VEGFR2 in all three cancer cell lines. Furthermore, compounds **6** and **7** demonstrated an inhibitory effect comparable to that of tivozanib, although this was observed only in the HT29 and A549 lines, respectively. These two derivatives were identified also as exhibiting the most pronounced anti-angiogenic properties and affinity for two binding sites of the primary transport protein in plasma, namely human serum albumin (HSA). To conclude, oxazolo[5,4-*d*]pyrimidin-7(6*H*)-imine **8** has been considered to be promising as a targeted anticancer agent.

**Figure 5:**
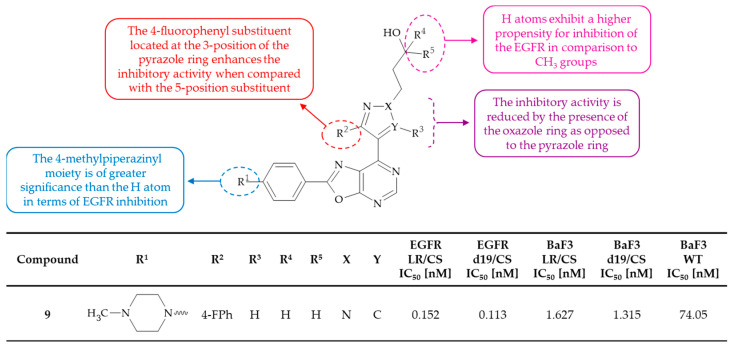
SAR study of oxazolo[5,4-*d*]pyrmidines as inhibitors of the EGFR and the EGFR C797S mutant.

Blank et al. prepared a library of 249 heterocyclic compounds, including some oxazolo[5,4-*d*]pyrmidines, which act as inhibitors of the EGFR and the EGFR C797S mutant [27]. The EGFR C797S mutation frequently occurs in conjunction with the EGFR L858R and/or the EGFR del19 mutations, e.g., L858R/C797S (LR/CS) mutations and/or del19/C797S (d19/CS) mutations. The in vitro inhibitory activity of the synthetized compounds was thus measured against an EGFR LR/CS and an EGFR d19/CS double mutant protein, using detection of chelation-enhanced fluorescence with improved sulfonamido-oxine (Sox) chromophore technology. The inhibitory effect of the compounds was also evaluated in a BaF3 assay against an EGFR wild-type (WT), an EGFR LR/CS mutant, and an EGFR d19/CS mutant. In the case of oxazolo[5,4-*d*]pyrimidine derivatives, the most potent compound **9** was identified as 3-[3-(4-fluorophenyl)-4-{2-[4-(4-methylpiperazin-1-yl)phenyl]oxazolo[5,4-*d*]pyrimidin-7-yl}-1*H*-pyrazol-1-yl]propan-1-ol. This compound was observed to inhibit an EGFR LR/CS and an EGFR d19/CS with IC_50_ values of 0.152 and 0.113 nM, respectively. Furthermore, it effectively inhibited the proliferation of BaF3 cells expressing an EGFR WT, an EGFR LR/CS, and an EGFR d19/CS. Based on SAR analysis, it is possible to establish general conclusions about the structural elements that have an impact on the activity of the oxazolo[5,4-*d*]pyrimidines (Figure 5). The introduction of the 4-methylpiperazine moiety in the phenyl ring at the C(2) position of the oxazolo[5,4-*d*]pyrimidine system has been shown to be beneficial for the inhibitory activity towards the EGFR, particularly the WT variant. Moreover, the pyrazole ring has been found to be a slightly superior substituent at the C(7) position when compared with the oxazole ring. In the context of the pyrazole ring substituents, it has been demonstrated that substitution at the 3 position resulted in a lower IC_50_ value than substitution at the 5 position. The IC_50_ values against an EGFR LR/CS were 5.2 and 196.8 nM, respectively. In conclusion, the invention provides potent inhibitors of EGFR comprising the L858R/C797S and/or del19/C797S mutations. It should be emphasized that an EGFR C797S point mutation has the potential to result in the development of resistance in cancer patients treated with osimertinib. Consequently, the compounds from the invention emerge as a potential treatment option for cancer patients who have developed resistance to other tyrosine kinase inhibitors, such as osimertinib.

### 2.2. Angiogenesis Inhibitors

Angiogenesis is the biological process by which new blood vessels are formed from existing ones. This process involves the degradation of the basement membrane, an increase in vascular permeability, and the proliferation, migration, and invasion of endothelial cells. Angiogenesis is an essential requirement for optimal cancer growth, as it enables the supply of nutrients and oxygen to cancerous cells. Angiogenesis plays a role in the progression of various types of cancer, including melanoma, breast cancer (BC), colorectal cancer (CRC), non-small cell lung cancer (NSCLC), and renal cell carcinoma (RCC) [28].

**Figure 6:**
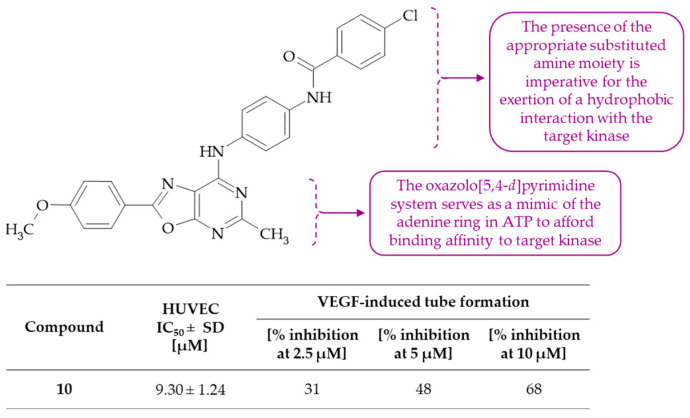
The structure and anti-angiogenic activity of oxazolo[5,4-*d*]pyrimidine-based benzamide.

Liu and colleagues proceeded to synthesize a novel oxazolo[5,4-*d*]pyrimidine derivative, which demonstrated potent, dose-dependent anti-angiogenic activity [29]. The application of virtual screening led to an improvement in the anti-angiogenic agent, **10**, which was identified as 4-chloro-*N*-(4-((2-(4-methoxyphenyl)-5-methyloxazolo[5,4-*d*]pyrimidin-7-yl)amino)phenyl)benzamide (Figure 6). The inhibitory efficacy of derivative **10** against HUVEC proliferation was found to reach a low IC_50_ value of 9.30 ± 1.24 µM. Moreover, it was demonstrated that compound **10** markedly inhibits the in vitro migration, chemotactic invasion, and capillary-like tube formation of HUVECs. It is noteworthy that treatment with oxazolo[5,4-*d*]pyrimidine **10** at concentrations of 0.1 µM, 1 µM, and 10 µM resulted in 83%, 56%, and 22% the number of migrated HUVECs, respectively, in comparison to the negative control group. Furthermore, the administration of compound **10** demonstrated a 31%, 48%, and 68% inhibition of VEGF-induced tube formation at 2.5 µM, 5 µM, and 10 µM, respectively. In an ex vivo model, derivative **10** was observed to effectively inhibit the formation of new microvessels from the rat aortic ring at level of 82%, 61%, and 29% at concentrations of 2.5 µM, 5 µM, and 10 µM, respectively. Results of Western blot analysis indicated that the downstream signaling of VEGFR2, including the phosphorylation of PI3K, ERK1/2, and p38 MAPK, was effectively inhibited by compound **10**. These decreases in the phosphorylated protein expression level, especially the phosphorylation of PI3K, suggested that oxazolo[5,4-*d*]pyrimidine **10** might be a potent inhibitor of the VEGFR2.

**Figure 7:**
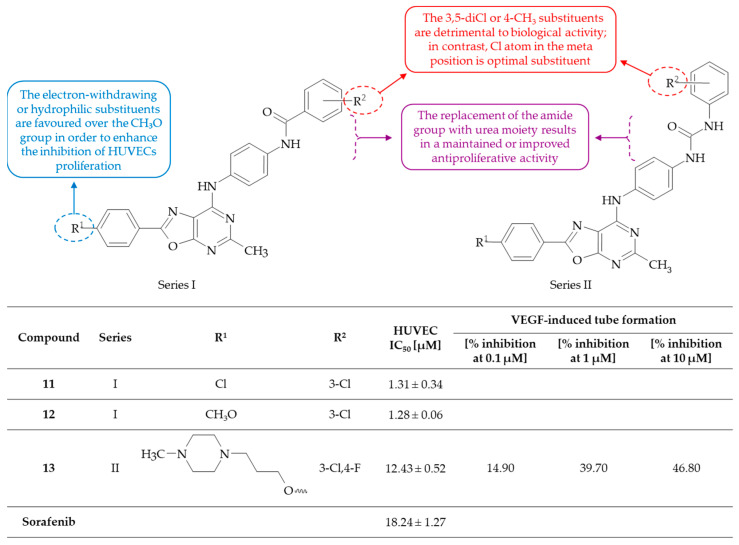
SAR study of oxazolo[5,4-*d*]pyrimidine-based ureas and amides as anti-angiogenic agents.

In accordance with the findings of Liu et al., Deng and colleagues presented the synthesis of a series of oxazolo[5,4-*d*]pyrimidine-based ureas and amides [30]. The antiproliferative and anti-angiogenic activities of the compounds were evaluated in comparison to sorafenib, a multi-kinase inhibitor. The kinase inhibitory effects of sorafenib have been demonstrated to impede cancer cell signaling, angiogenesis, and apoptosis [31]. The tested oxazolo[5,4-*d*]pyrimidine derivatives generally exhibited inhibitory activities against HUVEC in vitro. The most efficacious compounds, such as *N*-(4-{[2-(4-chlorophenyl)-5-methyloxazolo[5,4-*d*]-pyrimidin-7-yl]amino}phenyl)benzamide **11**, 3-chloro-*N*-(4-{[2-(4-methoxyphenyl)-5-methyloxazolo[5,4-*d*]-pyrimidin-7-yl]amino}phenyl)benzamide **12** and 1-(3-chloro-4-fluorophenyl)-3-{4-[(5-methyl-2-{4-[(4-methylpiperazin-1-yl)methoxy]phenyl}oxazolo[5,4-*d*]-pyrimidin-7-yl)amino]phenyl}urea **13**, demonstrated inhibition of the VEGF-induced HUVEC with IC_50_ values of 1.31 ± 0.34, 1.28 ± 0.06, and 12.43 ± 0.52 µM, respectively. In comparison with sorafenib, which exhibited an IC_50_ of 18.24 ± 1.27 µM, these activities were found to be more effective. The SAR analysis revealed that the phenyl ring in the C(2) position of the oxazolo[5,4-*d*]pyrimidine system substituted with a 4-Cl atom or a methylpiperazine moiety is preferred to the antiproliferative activity of the compounds of these series (Figure 7). The introduction of a 4-CH_3_O group to the phenyl ring has proven to be adverse to antiproliferative activity. Furthermore, the replacement of the 4-Cl atom with a 4-CH_3_O group led to a significant loss of VEGF-induced HUVEC inhibitory activity, with the exception of one compound, designated as **12**, which exhibited the best antiproliferative activity among the series. Moreover, the replacement of the amide linker with urea resulted in the maintenance or enhancement of antiproliferative activity. The optimal substituent was identified as a Cl atom at the meta position on the phenyl ring in substituent C(7) of the oxazolo[5,4-*d*]pyrimidine core. In particular, the incorporation of a thiazole ring instead of a phenyl ring resulted in outcomes that were deemed to be unsatisfactory. This finding suggests that the phenyl ring is essential for receptor binding. However, the 3,5-diCl and 4-CH_3_ substituents on the phenyl ring also exhibited no activity in the HUVEC proliferation test. In light of the findings, it was determined that among the most potent compounds, derivative **13** was selected for further biological evaluation. This decision was made due to its ability to inhibit the proliferation of HUVECs in a dose-dependent manner, as evidenced by the dose–response curve, which exhibited a more ‘S’-like profile in comparison to compounds **11** and **12**. In the course of the studies, compound **13** was found to reveal its capacity to reduce the VEGF-induced invasion by 14.90%, 39.70%, and 46.80%, respectively, at concentrations of 0.1, 1, and 10 µM. Furthermore, it was demonstrated that the compound exhibited a concentration-dependent inhibition on tube formation of HUVEC, as well as capillary sprouting from rat aorta rings. Preliminary mechanistic studies revealed that the anti-angiogenic activity of compound **13** was related to the suppression of protein kinase activation, by decreasing phosphoinositide 3-kinase (PI3K) and extracellular signal-regulated kinase 1/2 (ERK 1/2) phosphorylation. The results obtained thus far provide a strong rationale for the further investigation of this class of compounds with a view to designing improved molecules that target angiogenesis.

### 2.3. FGFR1 Inhibitors

Fibroblast growth factor receptor type 1 (FGFR1) is a member of the fibroblast growth factor receptor family, which is expressed on the cell membrane and regulates a number of cellular processes, including proliferation, differentiation, and survival. FGFR1 also plays a pivotal role in cell signaling and development. In cancers, it has been demonstrated that FGFR1 is deregulated by amplification, point mutation, or translocation. In particular, aberrant FGFR1 activation is associated with specific types of breast, lung, and bladder cancer [32].

**Figure 8:**
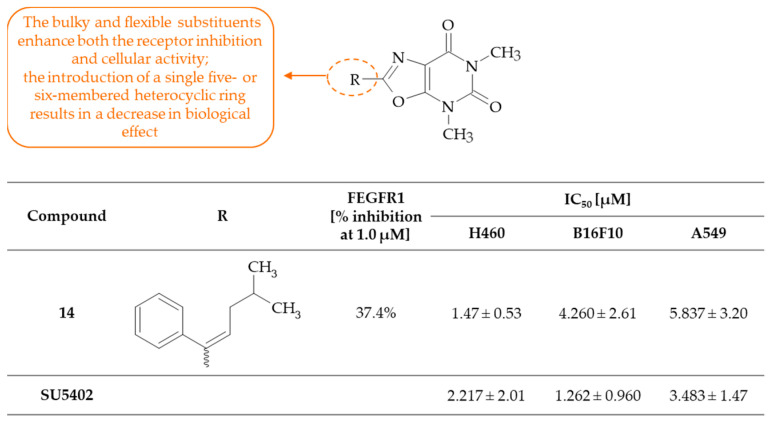
SAR study of 4,6-dimethyl-oxazolo[5,4-*d*]pyrimidine-5,7(4*H*,6*H*)-dione derivatives as anticancer agents with inhibitory activity against FEGFR1.

Ye and coworkers designed and synthetized the series of 5,7-dimethyl-oxazolo[5,4-*d*]pyrimidine-4,6(5*H*,7*H*)-dione derivatives, which were evaluated for their FGFR1-inhibition ability as well as cytotoxicity against three cancer cell lines (mouse melanoma B16F10 and human lung cancer H460 and A549) in vitro [33]. The reference drug was a potent multi-targeted receptor tyrosine kinase inhibitor, namely SU5402, with IC_50_ of 20 nM, 30 nM, and 510 nM for VEGFR2, FGFR1, and PDGF-Rβ, respectively [34]. The dose-dependent activity against the FGFR1 kinase was observed for most derivatives, among which compound **14**, i.e., (*E*)-2-(4-methyl-1-phenylpent-1-en-1-yl)-4,6-dimethyl-5*H*-oxazolo[5,4-*d*]pyrimidine-5,7(4*H*,6*H*)-dione, demonstrated a pronounced inhibition of FGFR1 (37.4% at 1.0 µM), reaching a comparable level of efficacy to the positive control SU5402. It is noteworthy that compound **14** also exhibited notable potency at a concentration of 0.1 µM and demonstrated enhanced selectivity towards FGFR1. This indicates that the compounds with substituent-containing stereo-structure groups exhibited superior activity compared to those with planar structures. It was observed that there was a notable reduction in inhibitory activity when the oxazolo[5,4-*d*]pyrimidine-5,7(4*H*,6*H*)-dione system was substituted at the C(2) position with a heterocyclic ring, such as furan, thiophen, or pyridine, suggesting that this group is too small to occupy the hydrophobic pocket or form a bond with FGFR1. Additionally, the large substituent was likely to form a stable interaction with the hydrophobic pocket, which may impede the movement of the compound side chain out of this region. This can be attributed to the space structure of the FGFR1 ATP pocket, which is sufficiently big to facilitate larger molecule integration and the formation of multiple bonds with these molecules. Furthermore, several compounds displayed good-to-excellent potency against tested cancer cell lines compared to SU5402. The results of the SAR analysis indicated that compounds with a stereo-structure groups exhibited greater efficacy than those lacking these substituents. Moreover, SAR demonstrated that the bulky and flexible groups may play a pivotal role in the regulation of kinase activity and cellular activity (Figure 8). Compound **14** was identified as exhibiting the most potent anticancer activity, with IC_50_ values of 5.472, 4.260, and 5.837 µM against the H460, B16F10, and A549 cell lines, respectively. These activities are comparable to those of the reference compound SU5402. It can be hypothesized that the greater number of hydrophobic groups present in derivative **14** may result in greater membrane permeability, in comparison to the other compounds in the series. In conclusion, the presented results suggest that 5,7-dimethyl-oxazolo[5,4-*d*]pyrimidine-5,7(4*H*,6*H*)-dione derivatives may serve as potential agents for the treatment of FGFR1-mediated cancers.

### 2.4. CB_2_ Receptor Neutral Antagonist

The cannabinoid type 2 (CB_2_) receptor is a component of the endocannabinoid system, which plays a role in a number of physiological processes. In particular, the CB_2_ receptor is expressed in the presence of active inflammation [35]. Recently, the CB_2_ receptor has been shown to be overexpressed in a wide range of human cancer tissues and to be associated with cancer progression. The CB_2_ receptor plays a role in the pathology of various cancers, including melanoma, non-small-cell lung cancer, colon cancer, and bladder cancer. The upregulation of CB_2_ in colon cancer has been associated with a poor prognosis for patients and an elevated proliferation rate of cancer cells [36].

**Figure 9:**
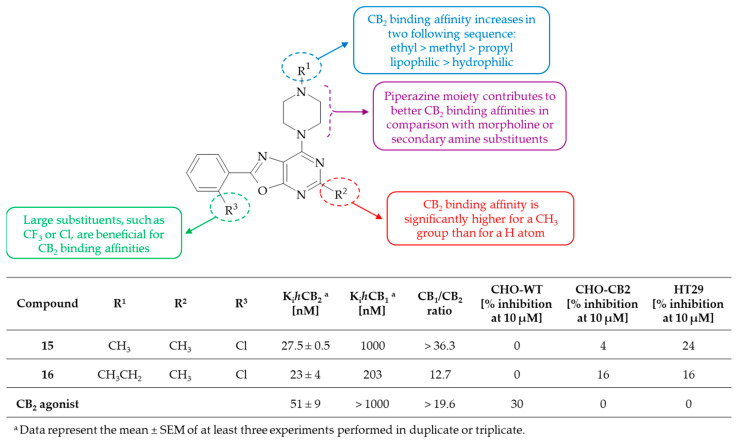
SAR study of oxazolo[5,4-*d*]pyrimidine derivatives with affinity for CB_1_/CB_2_ receptors.

Tuo et al. found that oxazolo[5,4-*d*]pyrimidine derivatives have an affinity for CB_1_/CB_2_ receptors [37]. Some of the compounds were identified as CB_2_ ligands with K_i_ values less than 1 µM. Notably, 2-(2-chlorophenyl)-5-methyl-7-(4-methylpiperazin-1-yl)oxazolo[5,4-*d*]pyrimidine **15** and 2-(2-chlorophenyl)-7-(4-ethylpiperazin-1-yl)-5-methyloxazolo[5,4-*d*]pyrimidine **16** show a strong affinity for CB_2_ receptor at nanomolar concentrations (Ki = 27.5 nM and 23 nM, respectively). These derivatives are significant selective CB_2_ ligands over CB_1_ receptors with selectivity index of SI > 36 and SI = 12.7 for oxazolo[5,4-*d*]pyrimidine **15** and **16**, respectively. Additionally, they behave as competitive neutral antagonists of the CB_2_ receptors because of a reduction in the agonist-induced inhibition of cAMP in Chinese hamster ovary cells, expressing CB_2_ receptors (CHO-CB_2_). The SAR analysis indicates that piperazine substituents at the C(7) position of the oxazolo[5,4-*d*]pyrimidine system improve the CB_2_ binding affinities of the compounds (Figure 9). Lipophilic piperazine substituents, such as methylpiperazine and ethylpiperazine, contribute to better CB_2_ binding affinity in comparison with a hydrophilic piperazine moiety such as acetylpiperazine. However, propylpiperazine substituents cause a significant decrease in CB_2_ affinity, which indicates steric limitations in receptor binding. On the other hand, the replacement of a H atom with the CH_3_ group at the C(5) position of the oxazolo[5,4-*d*]pyrimidine core resulted in a significant increase in the CB_2_ binding affinities of the compounds. These results indicate that the CH_3_ group plays a crucial role in binding affinity for CB_2_, probably due to hydrophobic interactions with the receptor. Moreover, the introduction of the CF_3_ group or Cl atom on the phenyl ring at the C(2) position of the oxazolo[5,4-*d*]pyrimidine scaffold causes an increase in CB_2_ binding affinities. Finally, compounds **15** and **16** were found to be more potent ligands for CB_2_ receptors than the reference CB_2_ agonist developed by Eli Lilly et al. (Ki = 51 nM) [38]. It should be emphasized that the introduction of an oxazolo[5,4-*d*]pyrimidine moiety instead of a 9*H*-purine ring improves CB_2_ binding affinities to be twice as potent. The results also indicated that the most potent derivatives, **15** and **16**, displayed no cytotoxic effect on tested cell lines, i.e., wild- type CHO, CHO-CB_2_, and colon cancer (HT29). The affinity toward the CB_2_ receptor of these compounds corresponds with the low cytotoxicity, probably due to their interaction with other targets. The low sensitivity of HT29 cancer cells to the synthesized compounds suggests that cannabinoid signaling is not decisive in their development. In summary, oxazolo[5,4-*d*]pyrimidine derivatives characterized with a Cl atom at the ortho position of the phenyl ring, a C(5)-substituted CH_3_ group, and a C(7)-substituted piperazine moiety can act as effective bioisosteres of purines in terms of CB_2_ binding with good selectivity over CB_1_ receptors (SI > 12).

### 2.5. AURKA Inhibitors

Aurora kinase A (AURKA) is a serine/threonine protein kinase that plays a pivotal role in mitosis. AURKA is involved in the maturation and separation of centrosomes, which in turn regulates spindle assembly and stability. The overexpression or mutations of AURKA result in errors in cell division and uncontrolled cell growth. The deregulation of AURKA has been associated with the pathogenesis of various cancers, including solid tumors and hematological malignancies. Consequently, AURKA has been identified as a potential target for cancer therapy [39].

**Figure 10:**
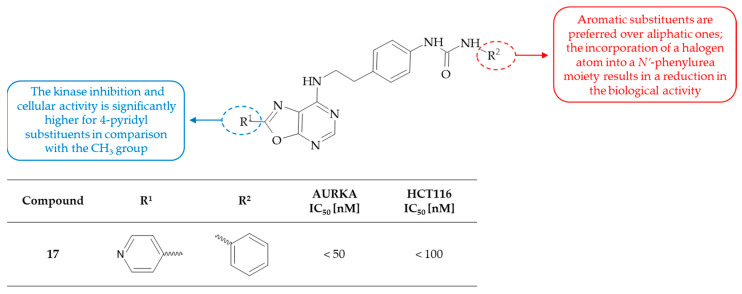
SAR study of oxazolo[5,4-*d*]pyrimidine derivatives as AURKA inhibitors.

Hsieh and colleagues identified a series of fused multicyclic compounds, including oxazolo[5,4-*d*]pyrimidine derivatives, which have the potential to inhibit the activity of Aurora kinase A [40]. The authors stated that the invention can be used to treat a range of cancers, including solid tumors of various organs, such as pancreatic, bladder, colorectal, breast, prostate, lung or renal, as well as hematological malignancies, such as acute myeloid leukaemia (AML), chronic myelogenous leukaemia (CML), acute lymphoblastic leukaemia (ALL), chronic lymphocytic leukaemia (CLL), Hodgkin’s disease (HD) and non-Hodgkin’s lymphoma (NHL). The cytotoxic activity against the human colorectal carcinoma cell line (HCT116) was determined through experimentation. The most active derivative among the series of oxazolo[5,4-*d*]pyrimidines was identified as compound **17**, i.e., *N*-[4-(2-{[2-(pyridin-4-yl)oxazolo[5,4-*d*]pyrimidin-7-yl]amino}ethyl}phenyl]-*N’*-phenylurea. This derivative was an in vitro inhibitor of AURKA with IC_50_ values between 1 and 50 nM. In addition, derivative **17** was active against HCT116 cancer cells in vitro with a cytotoxicity IC_50_ value lower than 100 nM. The SAR in this series indicated the significance of various pyridyl and phenyl substituents at the C(2) position of the oxazolo[5,4-*d*]pyrimidine system, since the methyl-substituted derivative was biologically inactive (Figure 10). In particular, the 4-pyridyl substituent was essential for maintaining activity, as demonstrated by the significantly superior kinase inhibition and cytotoxic activity of compound **17** in comparison with other derivatives. Moreover, the replacement of the 4-pyridyl substituent with a phenyl group resulted in the loss of the corresponding compound’s cytotoxic activity. Furthermore, the introduction of a halogen atom in the *N’*-phenylurea moiety of the substituent in the C(7) position of the oxazolo[5,4-*d*]pyrimidine core has been observed to result in a reduction in biological activity. It is notable that the compound, which contains a phenyl ring at the C(2) position and a *N’*-cyclopropylurea moiety, exhibits no activity in either the kinase or cytotoxic assay. This finding demonstrated the importance of the pyridyl group at the C(2) position and *N’*-phenylurea moiety at the C(7) position of the oxazolo[5,4-*d*]pyrimidine ring for interacting with AURKA. To conclude, the novel compounds may be applied in treating protein kinase-mediated cancers.

### 2.6. JAKs Inhibitors

Janus kinase 1 (JAK1) and Janus kinase 2 (JAK2) constitute a family of non-receptor protein tyrosine kinases that interact with a diverse array of cytokine receptors and hormones, including interleukins, interferons, growth hormones, erythropoietin, thrombo-poietin, and leptin. This results in the transmission of signals to cells, which influences gene expression and cellular responses [41]. The family of Janus kinases (JAKs) represents a primary mediator of inflammatory cytokines that phosphorylate and activate the signal transduction and activator of transcription (STAT) pathway. The involvement of JAK/STAT signaling in cell proliferation, stem cell maintenance, differentiation, and secretion of cytokines has been demonstrated to have a critical role in the induction of autoimmune diseases and cancerogenesis. Consequently, the abnormal activation of the JAK/STAT pathway has been identified as a factor that increases the risk of cancers, autoimmunity, and inflammatory diseases [42]. In particular, JAK1 is crucial in the progression of metastatic cancer [43].

**Figure 11:**
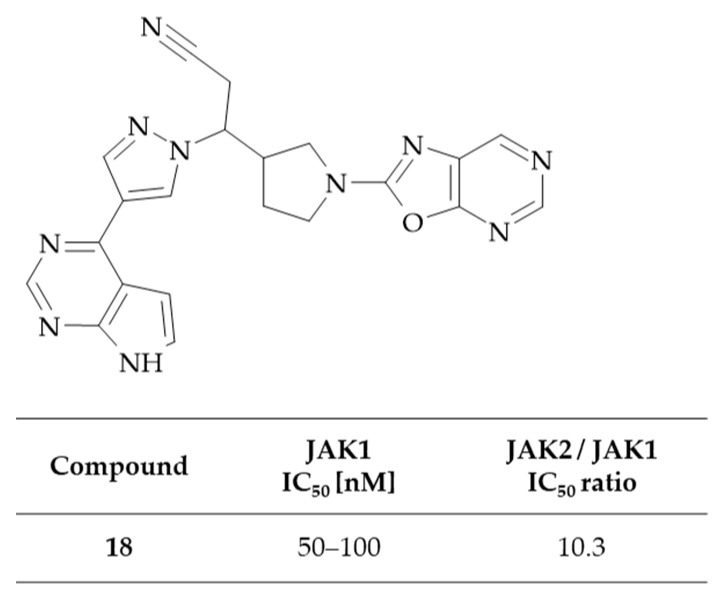
The structure and inhibitory activity against JAKs of oxazolo[5,4-*d*]pyrimidine-based nitrile.

In their patent, Rodgers et al. described *N*-(hetero)aryl-pyrrolidine derivatives as JAKs inhibitors, including selective JAK1 inhibitors [44]. The inhibitory activity of JAK targets expressed by baculovirus in insect cells was evaluated by measuring the phosphorylation of a biotinylated peptide. One of the compounds in the presented series comprises an oxazolo[5,4-*d*]pyrimidine system, specifically 3-(1-oxazolo[5,4-*d*]pyrimidin-2-ylpyrrolidin-3-yl)-3-[4-(7*H*-pyrrolo[2,3-d]pyrimidin-4-yl)-1*H*-pyrazol-1-yl]propanenitrile **18**. This derivative was subjected to assay conditions that accounted for an ATP concentration of 1 µM, resulting in an IC_50_ value of 50 to 100 nM for JAK1, which is 10.3 times higher than that observed for JAK2 (Figure 11). The lack of testing on more than one oxazolo[5,4-*d*]pyrimidine derivative precludes the possibility of conducting a SAR analysis for this class of compounds. However, the obtained results indicated that replacing the oxazolo[5,4-*d*]pyrimidine moiety with other oxazole-containing fused heterocyclic rings, such as oxazolo[5,4-*b*]pyridine, oxazolo[4,5-*c*]pyridine, or benzoxazole, enhanced JAK1 inhibitory activity. Consequently, the most notable enhancement in selectivity between kinases was observed upon the introduction of a 3-cyano-4-iodopyridin-2-yl and 2,6-dichloropyridin-3-yl substituents in place of the oxazolo[5,4-*d*]pyrimidine core (JAK2/JAK1 IC_50_ ratios were 188 and 132.5, respectively). In conclusion, the compounds resulting from the presented invention have the potential to be useful in the treatment of diseases associated with the JAK/STAT pathway, including inflammatory and autoimmune disorders, as well as cancer.

### 2.7. NAE Inhibitors

NEDDylation is a biochemical process of post-translational protein modification through three-step enzymatic cascades. The initial stage of NEDDylation is catalyzed by the NEDD8-activating enzyme (NAE), which is an ATP-dependent activating enzyme involved in the addition of the ubiquitin-like protein, namely neural precursor cell-expressed developmentally downregulated protein 8 (NEDD8), to a target molecule [45]. As a case in point, the substrates of NEDDylation are the cullin-RING ubiquitin ligases. These substrates regulate a number of key cellular processes by promoting the process of ubiquitination and subsequent degradation of various regulatory proteins. This, in turn, activates pathways that promote cancer cell growth and survival. Notably, the NEDDylation process and/or its substrates exhibit abnormal activation or overexpression in diseases such as cancer, liver dysfunction, and metabolic or neurodegenerative disorders [46]. In the context of enzymes associated with the NEDDylation modification, NAE has emerged as a particularly promising target for the development of anticancer drugs [47].

**Figure 12:**
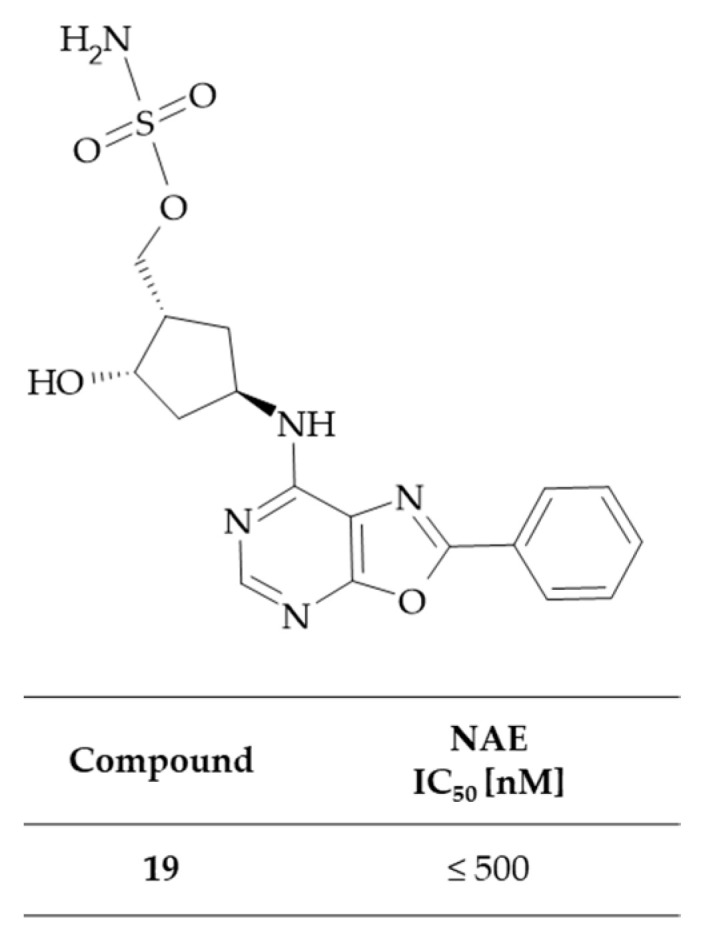
The structure and inhibitory activity against NAE of oxazolo[5,4-*d*]pyrimidine-based sulfamate.

Claiborne et al. reported the synthesis of a series of sulfamate derivatives as potential NAE inhibitors [48]. These compounds were characterized by the presence of various nitrogen-containing heteroaryl rings, including 6-membrane rings such as pyridine, pyrimidine, and 1,3,3-triazine, which were optionally fused to 5- or 6-membered rings, such as benzene, imidazole, pyrrole, or oxazole rings. It was found that these compounds were effective inhibitors of El activating enzymes, particularly NAE. Consequently, they may have therapeutic potential in the treatment of disorders of cell proliferation, including cancers and inflammatory disorders. A homogeneous time-resolved fluorescence (HTRF) assay with a recombinant human NEDD8-activating enzyme was used to evaluate the inhibition of NAE by the tested compounds. In the present invention, a single derivative was identified as containing the oxazolo[5,4-*d*]pyrimidine system. This compound, designated as {(lS,2S,4R)-2-hydroxy-4-[(2-phenyloxazolo[5,4-*d*]pyrimidin-7-yl)-amino]cyclopentyl}methyl sulfamate **19**, demonstrated a potent IC_50_ value of 500 nM or less against NAE (Figure 12). The findings of this study therefore suggest that sulfamate derivatives have the potential to be effective NAE inhibitors in the treatment of cancer. This hypothesis is further evidenced by the fact that two of the sulfamate derivatives, namely MLN4924 (Pevonedistat) and TAS4464, are currently undergoing clinical trials for anticancer applications [47].

### 2.8. HGPRT Inhibitors

Hypoxanthine-guanine phosphoribosyltransferase (HGPRT) is an enzyme that plays a pivotal role in the purine salvage pathway. Within this pathway, it catalyzes the reaction of hypoxanthine and guanine with 5-phosphoribosyl-1-pyrophosphate (PRPP) to form their respective nucleotides. Consequently, this enzyme ensures the supply of essential purine nucleotides for DNA synthesis. It has been observed that HGPRT is subject to upregulation in cancer cells. Recent studies have identified a novel function for HGPRT in cancer biology, suggesting its potential as a cancer biomarker [49]. It is also postulated that HGPRT may have an additional role in the regulation of cancer proliferation [50]. The significant surface localization of HGPRT within certain cancer cells suggests its potential application as a surface antigen in targeted immunotherapy [51]. However, further study is required to elucidate the role of HGPRT in cancer.

**Figure 13:**
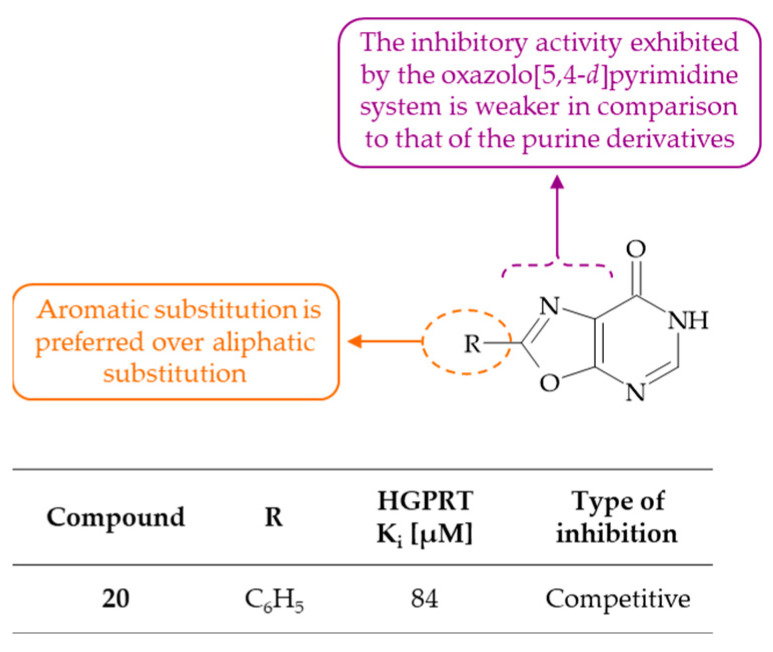
SAR study of oxazolo[5,4-*d*]pyrimidine derivatives as HGPRT inhibitors.

Jadhav and colleagues conducted a series of experiments in which they tested a range of purines and purine derivatives, including oxazolo[5,4-*d*]pyrimidine and thiazolo[5,4-*d*]pyrimidine, as potential inhibitors of human erythrocytic hypoxanthine-guanine phosphoribosyltransferase [52]. The oxazolo[5,4-*d*]pyrimidines were found to be competitive HGPRT inhibitors. Among these, the highest potency was demonstrated by 2-phenyloxazolo[5,4-*d*]pyrimidine-7-one **20** (K_i_ = 84 µM). In comparison to purines, oxazolopyrimidines and thiazolopyrimidines exhibited weaker affinity for HGPRT. The study enabled the establishment of further structural requirements for inhibition (Figure 13). It was found that the aromatic substituent at the C(2) position of the oxazolo[5,4-*d*]pyrimidine core is more favored than an aliphatic one. Furthermore, a comparison of derivatives with different heterocyclic cores showed that the oxazolo[5,4-*d*]pyrimidine or izothiazolo[5,4-*d*]pyrimidine system generally leads to a decrease in activity compared to the purine derivatives. Consequently, the exploration of variously substituted purine scaffolds appears to be a rational approach to the development of potent, specific HGPRT inhibitors.

### 2.9. Apoptosis Inducers

#### 2.9.1. Antiapoptotic Proteins Inhibitors

Apoptosis is a process of programmed cell death that can be initiated by intrinsic (i.e., mitochondrial) or extrinsic (i.e., receptor) signaling pathways. Members of the B-cell lymphoma 2 (BCL-2) protein family serve as crucial regulators of apoptotic cell death, exhibiting both pro- and anti-apoptotic activities. Given that the overexpression of pro-survival members of BCL-2 protein family or the reduction of its pro-apoptotic members results in the inhibition of apoptosis, both are frequently detected in various types of cancers. BCL-2 is an example of a pro-survival (i.e., anti-apoptotic) protein, the activity of which is predominantly observed at the mitochondrial level by preventing cytochrome c release. The use of BCL-2 inhibitors results in a decrease in protein production, thereby blocking the anti-apoptotic defense mechanism of cancerous cells. This subsequently releases pro-apoptotic proteins, which then induce apoptosis and thus achieve anticancer effects [53,54].

**Figure 14:**
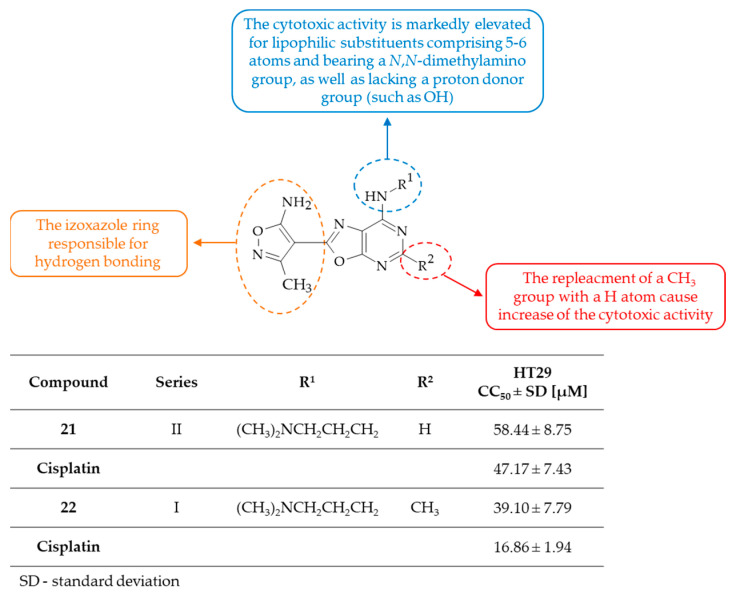
SAR study of isoxazole-substituted oxazolo[5,4-*d*]pyrimidines as anticancer agents.

The two series of oxazolo[5,4-*d*]pyrimidine derivatives, which exhibited BCL-2 inhibitory activity, were synthesized by Sochacka-Ćwikla et al. [55,56]. The designed compounds were characterized by the presence of a favorable isoxazole substituent at the C(2) position of the condensed heterocyclic system, along with aliphatic-amino chains at the C(7) position. In the course of the research, a derivative from series I with a methyl group at the C(5) position and a pentylamino substituent at the C(7) position was found to completely block the synthesis of BCL-2 protein. Furthermore, a compound from series II with an H atom at the C(5) position and a 3-(*N*,*N*-dimethylamino)propylamino substituent at the C(7) position, namely **21**, was found to decrease the BCL-2 level in the HT29 cell line. The in vitro evaluation of the synthetized compounds was conducted for their cytotoxic activity against a panel of human cancer cell lines, including A-431 epidermal, A549 lung, MCF7 breast, HT29 primary colon, and LoVo metastatic colon, as well as one mouse cancer cell line, i.e., L1210 leukemia. The reference drugs employed in these studies were 5-fluorouracil (5-FU) and/or cisplatin, as evidenced by their remarkable anticancer activity against the tested human cell lines [57,58]. Of particular note was the finding that the primary colon cancer (HT29) cell line exhibited the greatest sensitivity to the influence of both series of oxazolo[5,4-*d*]pyrimidines. The most cytotoxically active derivatives were **22**, with methyl group at the C(5) position and 3-(*N*,*N*-dimethylamino)propylamino substituent at the C(7) position, and **21**, from series I and II, respectively. It is intriguing that the signaling pathways induced by the **22** did not involve the caspase, NFκB, BCL-2, and Fas pathway despite its very potent biological activity. However, compound **22** increased the expression of components of mitogen-activated protein kinase pathways (MAPK) such as JNK, p38α, and p38β. Further studies of the mechanism of action of oxazolo[5,4-*d*]pyrimidines have indicated that this process involves the apoptosis of cancerous cells. Moreover, derivative **21** has been demonstrated to have the ability to inhibit P-glycoprotein and to inhibit the migration of HT29 cells. Theoretical research using molecular docking has identified oxazolo[5,4-*d*]pyrimidine derivatives from series II as potent and selective inhibitors of human VEGFR-2. Subsequent SAR analysis of compounds from series I and II revealed that the activity of the compounds is contingent on the size of the substituent in position C(7), and the presence of appropriate functional groups therein (Figure 14). It was found that compounds bearing aliphatic substituents consisting of 1 to 3 carbon atoms, as terminal hydrophobic moieties, were less preferred biologically and exhibited relatively weak or a lack of cytotoxic activity against all selected cancer lines. The presence of a proton donor group at the end of the substituent in compounds was generally responsible for the lack of cytotoxic activity. In contrast, it was revealed that incorporation of 3-(*N*,*N*-dimethylamino)propylamino or a pentylamino moiety, with a length of approximately six atoms, into the C(7) position of the oxazolo[5,4-*d*]pyrimidine system resulted in a significant increase in the cytotoxic activity against the HT29 cell line of both series of compounds. Further comparison of the cytotoxic activity against the HT29 cell line among series I and II revealed that the replacement of the CH_3_ group by an H atom at position C(5) of the oxazolo[5,4-*d*]pyrimidine core resulted in an increase in activity compared to the reference drug, i.e., cisplatin. It is noteworthy that the 5-methyl analog of compound **21** (i.e., **22**) had activity against HT29 with a half maximal cytotoxic concentration (CC_50_) equal to 39.10 ± 7.79 µM in comparison with cisplatin with CC_50_ = 16.86 ± 1.94 µM (estimated on the basis of data taken from ref. [55]). This indicates that compound **21** has statistically equal cytotoxic activity as cisplatin and its 5-methyl derivative **22** is statistically less active than cisplatin. Moreover derivative **21** was less toxic to healthy human cells (such as normal human dermal fibroblasts (NHDF)) than the reference drug. To conclude, the appropriate combination of isoxazole and oxazolo[5,4-*d*]pyrimidine rings may be an important scaffold in the development of potentially effective anticancer agent.

#### 2.9.2. Caspase Cascade Activators

Caspases represent a family of cysteine proteases for which a significant role in the signaling cascade leading to apoptosis has been demonstrated. These can be categorized into two distinct classes: effector caspases (e.g., caspase-3, -6, and -7) and initiator caspases (e.g., caspase-2, -8, -9, and -10). Within the extrinsic apoptosis pathway, the Death-Inducing Signalling Complex (DISC) has been identified as a critical element in the process of caspase dimerization, which is considered to be a pivotal step for activating initiator caspases 8 and 10 [59]. In contrast, the intrinsic pathway is primarily activated by cellular stresses including DNA damage or metabolic stress. A deregulation of the activation of the caspase cascade, in addition to high expression of caspase inhibitors, has been observed in cancerous cells. This has been shown to contribute to the development of resistance to cell death and subsequent cancer progression. Recent therapeutic applications have involved the activation of individual caspases and the repression of natural inhibitors of caspases [60].

**Figure 15:**
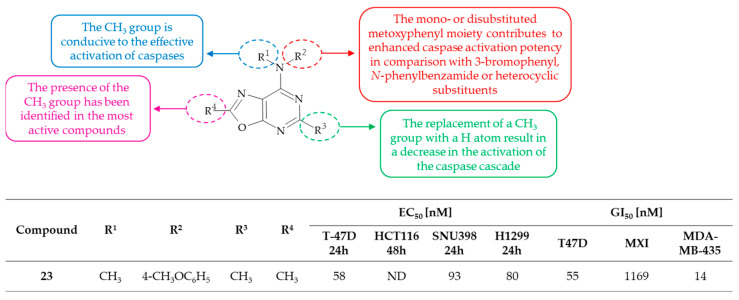
SAR study of oxazolo[5,4-*d*]pyrimidin-7-amine derivatives as activators of caspases.

In the course of their research, Cai and colleagues synthesized a series of *N*-aryl-isoxazolopyrimidin-4-amines and related compounds, including oxazolo[5,4-*d*]pyrimidin-7-amines [61]. These compounds were designed to act as activators of caspases and inducers of apoptosis. The invention also relates to the use of these compounds as therapeutically effective anticancer agents. The activation of caspases was investigated in four human solid cancer cell lines: breast cancer T-47D, hepatocellular carcinoma SNU398, colon carcinoma HCT116, and lung cancer H1299, with using *N*-Ac-DEVD-*N’*-ethoxycarbonyl rhodamine 110 as a fluorogenic substrate. Moreover, the anticancer activity of the synthesized compounds was evaluated against a range of cell lines, including human breast cancer (T-47D, MX-I and MDA-MB-435), human sarcoma (MES-SA), multi-drug resistant human sarcoma (MES-SA/ADR), murine leukemia (P388) and multidrug resistant murine leukemia (P388ADR). The findings demonstrated that among oxazolo[5,4-*d*]pyrmidines, derivative **23**, i.e., *N*-(4-methoxyphenyl)-*N*,2,5-trimethyloxazolo[5,4-*d*]pyrimidin-7-amine, functions as an activator of the caspase cascade in various cancer cell lines, exhibiting half maximal effective concentration (EC_50_) values ranging from 58 to 93 nM. This compound was also found to inhibit the proliferation of T-47D and MDA-MB-435 cell lines with 50% growth inhibition concentrations (GI_50_) of 55 and 14 nM, respectively. The caspase activation potency of other oxazolo[5,4-*d*]pyrimidine derivatives was significantly lower, with EC_50_ value equal or higher than 613 nM. Subsequent SAR analysis indicated that the presence of the 4-CH_3_O, 2,5-diCH_3_O or 3,5-diCH_3_O substituted aniline moiety at the C(7) position of the oxazolo[5,4-*d*]pyrimidine system were essential for the activation of the caspase cascade (Figure 15). It was demonstrated that the activity was most effective when this moiety contained the methyl group on the nitrogen atom. Conversely, the replacement of the CH_3_ group with a H atom at the C(5) position resulted in a reduction in activity. It is noteworthy that the analog of compound **23**, which possesses an isoxazolo[5,4-*d*]pyrimidin-4-amine core, has been identified as the most potent caspase cascade activator and inducer of apoptosis in solid cancer cells. Of particular significance is the finding that this derivative exhibits comparable activity in the inhibition of cell proliferation to that of MES-SA or P388 and their corresponding multi-drug resistant cell lines. Therefore, compounds of this invention have the potential to induce cell death in a range of clinical conditions characterized by uncontrolled growth and spread of abnormal cells.

## 3. Oxazolo[5,4-*d*]pyrimidines as Anticancer Agents with Undefined Mechanism of Action

**Figure 16:**
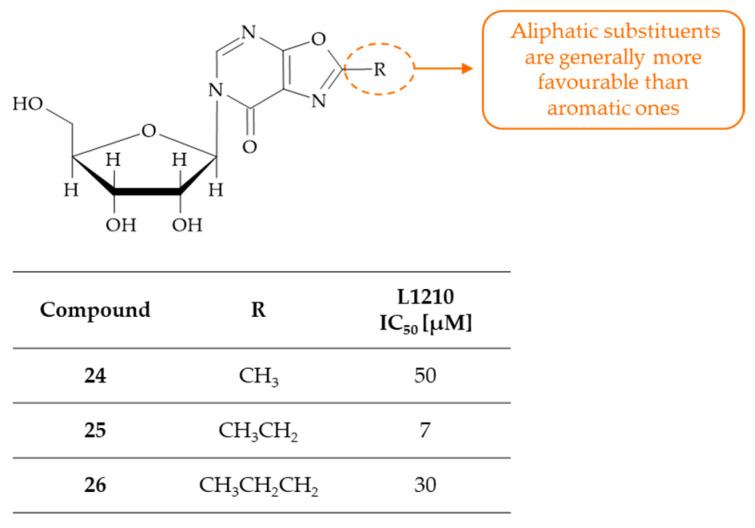
SAR study of 2-substituted 6-(β-D-ribofuranosyl)oxazolo[5,4-*d*]pyrimidin-7-ones as anticancer agents.

In 1974 Patil, Wise, Townsend, and Bloch described the synthesis and biological activity of selected 2-substituted 6-(β-D-ribofuranosyl)oxazolo[5,4-*d*]pyrimidin-7-ones as pyrimidine antagonists [62]. Among a series of compounds being evaluated for their inhibitory activity against leukemia L1210 cells in vitro, only derivatives substituted at the C(2) position with methyl (**24**), ethyl (**25**), or propyl (**26**) groups markedly inhibited the growth of cells. The IC_50_ values for the L1210 cell line were found to be 50 µM, 7 µM, and 30 µM for compounds **24**, **25**, and **26**, respectively. On the other hand, derivatives bearing a phenyl ring as a substituent at the C(2) position showed no substantial biological activity. In the in vivo evaluation, it was found that compound **24** exhibited significant activity against leukemia L1210. This was at a dose of 200 mg/kg/day × 5, which resulted in a 31% increase in the life span of the cancer-bearing mice. Subsequent to a SAR analysis encompassing both in vitro and in vivo experiments, it was deduced that the most optimal substituent in the C(2) position of the oxazolo[5,4-*d*]pyrimidine system is an aliphatic group with a length of between 1 and 3 atoms (Figure 16). Conversely, the incorporation of aromatic substituents at the C(2) position has been demonstrated to result in a reduction in anticancer activity. Overall, the compounds presented herein can be considered as analogs of a pyrimidine ring substituted with a cyclic moiety at positions 5 and 6.

**Figure 17:**
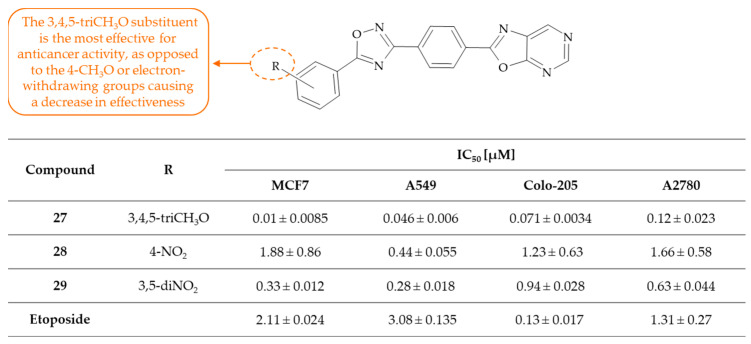
SAR study of 1,2,4-oxadiazole-linked 4-oxazolo[5,4-*d*]pyrimidine derivatives as anticancer agents.

Perupogu and colleagues conducted a series of 1,2,4-oxadiazole-linked 4-oxazolo[5,4-*d*]pyrimidine derivatives with anticancer activity [63]. 1,2,4-Oxadiazoles represent a significant class of five-membered ring-containing heterocyclic compounds, which are important building blocks for a variety of natural compounds with diverse pharmacological properties. On the other hand, oxazolo[5,4-*d*]pyrimidines are a unique class of fused heterocyclic compounds, which also possess a broad spectrum of biological activities. In view of the aforementioned information, a new series of 1,2,4-oxadiazole-linked 4-oxazolo[5,4-*d*]pyrimidine derivatives was designed and synthesized by the authors. The biological evaluation included the assessment of inhibitory activity towards four human cancer cell lines, namely breast cancer (MCF7), lung cancer (A549), colon cancer (Colo-205), ovarian cancer (A2780), and etoposide used as a positive control. Etoposide is a chemotherapy used in a treatment for a number of different cancer types [64]. The results demonstrated that the tested compounds exhibited moderate to excellent activity against all of the cell lines. Of particular note are derivatives **27**, identified as 2-(4-(5-(3,4,5-trimethoxyphenyl)-1,2,4-oxadiazol-3-yl)phenyl)oxazolo[5,4-*d*]pyrimidine, and **28**, i.e., 2-(4-(5-(4-nitrophenyl)-1,2,4-oxadiazol-3-yl)phenyl)oxazolo[5,4-*d*]pyrimidine, and **29**, namely 2-(4-(5-(3,5-dinitrophenyl)-1,2,4-oxadiazol-3-yl)phenyl)oxazolo[5,4-*d*]pyrimidine, which were found to be more potent than the positive control. IC_50_ values were found to range from 0.01 ± 0.0085 to 1.23 ± 0.63 µM. More detailed data are presented in Figure 17. A preliminary SAR study indicated that the 3,4,5-triCH_3_O group on the phenyl ring showed the highest anticancer activity. Conversely, 3,5-diCH_3_O and 4-CH_3_O substituents exhibited a reduction in anticancer activity across all tested cell lines. Furthermore, the replacement of the 4-CH_3_O group with either 4-Cl or 4-Br groups resulted in moderate activity. Interestingly, the presence of 4-NO_2_ and 3,5-diNO_2_ substituents was observed to result in a slightly enhancement in activity. The 4-CH_3_ group also exhibited notable activity. In summary, the encouraging results provide a basis for the continuation of research into the most potent molecules regarding their mechanism of action.

**Figure 18:**
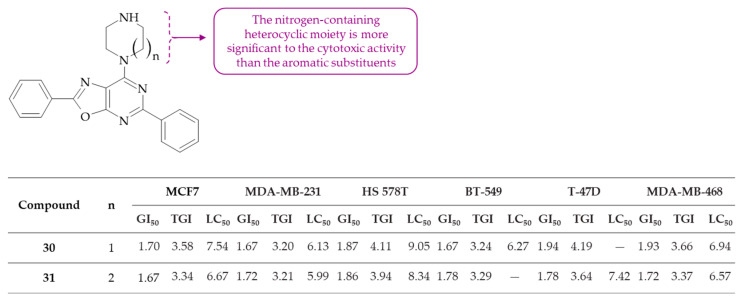
SAR study of oxazolo[5,4-*d*]pyrimidine derivatives as anticancer agents.

The series of oxazolo[4,5-*d*]pyrimidines and the two oxazolo[5,4-*d*]pyrimidine derivatives were synthesized by Velihina et al. [65]. The obtained compounds were then subjected to testing against a range of breast cancer cell lines from the NCI subpanel (namely, MCF7, MDA-MB-231, T-47D, HS 578T, BT-549 and MDA-MB-435) in both one-dose and five-dose assays. According to the GI_50_ of cell lines and to statistically significant differences in cytostatic activity (TGI), it can be stated that both oxazolo[5,4-*d*]pyrimidines, i.e., 2,5-Diphenyl-7-(piperazin-1-yl)oxazolo[5,4-*d*]pyrimidine **30** and 7-(1,4-diazepan-1-yl)-2,5-diphenyloxazolo[5,4-*d*]pyrimidine **31**, have been shown to have the same potent anticancer effect (Figure 18). In this study, the authors also postulate a hypothesis concerning their mechanism of action, proposing that it involves the induction of cell apoptosis or the inhibition of DNA replication. This is based on the COMPARE analysis data, which revealed that derivative **30** exhibited a remarkably high correlation with tamoxifen, a selective estrogen receptor modulator [66], and a high correlation with aclarubicin, a dual topoisomerase inhibitor [67]. However, it should be emphasized that this correlation has not been substantiated through any experimental assay. Statistical analysis indicates that compounds **30** and **31** have been shown to demonstrate equivalent cytotoxic activity, irrespective of the substituent at the C(7) position of oxazolo[5,4-*d*]pyrimidine core. It can thus be postulated that the heterocyclic fragment containing a nitrogen atom plays a significant role in their efficacy. A comparison of these results with other work by Velihina and colleagues is indicated, wherein the other two oxazolo[5,4-*d*]pyrimidines were obtained [68]. Importantly, these compounds differed from derivatives **30** and **31** only in terms of a substituent at the C(7) position, which was a phenyl or 4-(methoxycarbonyl)phenyl ring. Moreover, they were found to be inactive against the tested cancer cell lines, including breast cancer (MDA-MB-231), colon cancer (HCT116) and ovarian cancer (OVCAR-3). In view of the findings, it can be concluded that the presence of an aromatic substituent at the C(7) position is not conducive to biological activity. Consequently, the potential for molecules bearing a piperazine moiety as effective anticancer agents is a highly promising area for further investigation.

## 4. Conclusions

**Figure 19:**
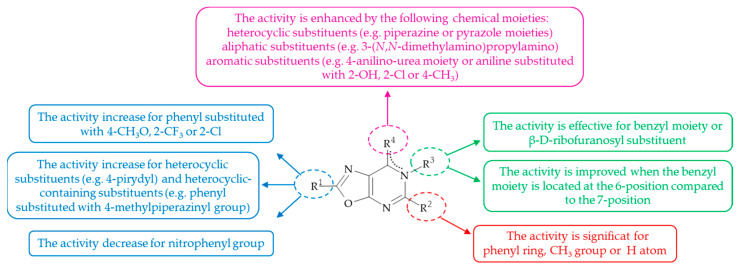
SAR of oxazolo[5,4-*d*]pyrimidines for anticancer activity. The dashed line represents a delocalized bond.

The oxazolo[5,4-*d*]pyrimidine scaffold has emerged as a promising candidate in the field of anticancer drug discovery. This is attributable to its chemical structure, which displays notable similarity to that of naturally occurring purine bases, and the range of its significant biological activities. The present review has demonstrated the capacity of oxazolo[5,4-*d*]pyrimidines to modulate multiple molecular pathways involved in cancer cell proliferation, survival, and metastasis, thus establishing them as versatile agents for the treatment of different types of cancers. The mechanism of action of these compounds involves the inhibition of various receptors, non-receptor protein kinases, angiogenesis, and other enzymes, such as NAE and HGPRT. The receptors that have been identified as their target include VEGFR2, EGFR, FGFR1, and CB_2_, while the non-receptor protein kinases that have been implicated are JAKs and AURKA. Furthermore, the oxazolo[5,4-*d*]pyrimidine derivatives have been demonstrated to function as apoptosis inducers, encompassing both antiapoptotic protein inhibitors and caspase cascade activators. The results of the study highlighted the potential of structural modifications to optimize the biological activity of these compounds. A preliminary investigation of the chemical properties of the oxazolo[5,4-*d*]pyrimidine system has revealed its ability to functionalize at four primary reactive sites, specifically at the 2, 5, 6, and 7 positions (Figure 19). SAR analysis indicates that the presence of a variety of substituents could result in effective anticancer activity. Furthermore, the use of SAR analysis and molecular docking studies facilitates the exploration of the significant interactions between oxazolo[5,4-*d*]pyrimidine derivatives and their molecular targets. Consequently, these findings offer a promising perspective for the development of a selective anticancer pharmacophore in the near future. To conclude, further investigation into the structural characteristics of FDA-approved anticancer drugs, along with the identification of relevant cancer biomarkers, has the potential to enhance the therapeutic efficacy of oxazolo[5,4-*d*]pyrimidines. The continuation of research and clinical trials is imperative to comprehensively elucidate their pharmacological profile and transition their potential into effective anticancer therapy.

## Data Availability

Not applicable.
